# Genome sequencing reveals extraordinary cephalic horns in the Madagascan dung beetle genus *Helictopleurus* (Coleoptera, Scarabaeinae): insight from a revision of *fungicola* species group

**DOI:** 10.3897/zookeys.1033.63527

**Published:** 2021-04-22

**Authors:** Michele Rossini, Olivier Montreuil, Vasily Grebennikov, Sergei Tarasov

**Affiliations:** 1 Finnish Museum of Natural History (LUOMUS), University of Helsinki, Pohjoinen Rautatiekatu 13, Helsinki , 00014 , Finland University of Helsinki Helsinki Finland; 2 UMR 7179 MNHN/CNRS, MECADEV, Muséum National d’Histoire Naturelle, Entomologie , CP 50, 45 rue Buffon, 75231 Paris cedex 05, France Muséum National d’Histoire Naturelle Paris France; 3 960 Carling Avenue, Ottawa, ON, K1A 0C6, Canada Unaffiliated Ottawa Canada

**Keywords:** dung beetles, *fungicola* species group, *Heterosyphus*, Madagascar, male horns, mitogenomics, Scarabaeinae

## Abstract

In this study, we test and corroborate the phylogenetic position of *Heterosyphus* within *Helictopleurus* using mitogenomes and nuclear loci. Our recent samplings revealed that males of the former *Heterosyphussicardi* Paulian, 1975 (today under *Helictopleurus* d’Orbigny, 1915) have extraordinary bilateral clypeal horns which are exclusive within the genus. We provide a taxonomic review of the *fungicola* species group of *Helictopleurus* and discuss the systematic position of *H.sicardi* within the group. The male phenotype of *H.sicardi* is described and photographs of the body and genitalia of the members of the *fungicola* group are given, as well as a diagnostic key to species of the group. *Helictopleurusfungicolapeyrierasi* is considered to be a distinct species within the genus (*H.peyrierasi***stat. rest.**). *Helictopleuruspluristriatus* d’Orbigny, 1915 **syn. nov.** is established as a junior synonym of *H.fungicola* (Fairmaire, 1899).

## Introduction

The dung beetle tribe Oniticellini (Coleoptera, Scarabaeinae) was represented by two endemic genera in Madagascar, namely *Helictopleurus* d’Orbigny, 1915 and *Heterosyphus* Paulian, 1975. With 68 known species and subspecies, *Helictopleurus* was thought to have males with only simple cylindrical horns or carina on the head, which are common across various dung beetle lineages. At the same time, the monotypic and extremely rare *Heterosyphus* was thought to be hornless. Previous phylogenetic analysis of Madagascan Oniticellini (Wirta et al. 2008) revealed a nested position of *Heterosyphus* within *Helictopleurus*; following these results Philips (2016) suggested the synonymy of *Heterosyphus* with *Helictopleurus*.

*Helictopleurussicardi*, the former member of the monotypic genus *Heterosyphus* (Paulian 1975), has been known so far by only four females and one hornless male from the northern Madagascar (Montagne d’Ambre) (see Material examined). Recent sampling of forest leaf litter yielded new specimens of this rare species, whose biology is still enigmatic. Two of those specimens are males with two spectacularly long horns that arise from the lateral sides of the clypeus. This polymorphism in males – presence vs. absence of horns – is common among dung beetles. Nonetheless, the bilateral clypeal horns observed in *H.sicardi* are unique within *Helictopleurus* and rare in other genera of the tribe Oniticellini and its sister, the tribe Onthophagini. It is noteworthy that similar bilateral clypeal horns occur in more distant dung beetle lineages such as, for example, the genera *Heliocopris* Hope, 1837 and *Bubas* Mulsant, 1842.

Thus, considering the exclusive phenotype of *H.sicardi* and its previous placement in a separate genus, we test the phylogenetic position of this species within *Helictopleurus* using mitogenomic data and nuclear loci. Our 19-gene phylogenetic analysis of *Helictopleurus* and other genera from the tribe Oniticellini corroborates the results of Wirta et al. (2008) by supporting the synonymy of *Heterosyphus* with *Helictopleurus*. Both morphological and molecular evidence suggest that *H.sicardi* is a member of the *fungicola* species group (*sensu*Montreuil 2005a) of *Helictopleurus*. We describe the male phenotype of *H.sicardi*, discuss the taxonomy and systematic position of the *fungicola* species group, reconsidering the status of *H.fungicolapeyrierasi* and proposing *H.pluristriatus* as a junior synonym of *H.fungicola*.

## Material and methods

### Material deposition

Voucher specimens and type material analyzed throughout the study are deposited in the following institutes:

**MNHN**Muséum national d’Histoire naturelle, Paris;

**MZHF** Finnish Zoology Museum of Natural History (LUOMUS), Helsinki (S. Tarasov, J. Mattila).

### Morphological examination

The external morphology, along with the anatomy of the male and female genitalia of a total of 39 *Helictopleurus* species currently assigned to seven of the nine species groups (Lebis 1960; Montreuil 2005a) were examined. The identification of the specimens was carried out by comparison with the name-bearing type material. Following the methodology of Tarasov and Génier (2015), at least one male and one female per species were completely disarticulated for a comprehensive scrutiny of their morpho-anatomy. Body parts were subsequently washed with distilled water and stored on tissue culture plates with glycerol. Male and female genitalia were cleaned in the KOH solution before being stored in glycerol, while hindwings were placed in glycerol after dissection.

Morphological study was performed under a Leica S9D stereomicroscope. Habitus photographs were taken with a Canon EOS 5D camera and a Canon MP-E 65mm, f/2.8, 1–5× macro lens, using the Cognisys Stackshot automated system; male genitalia were photographed with a Nikon SMZ25 stereomicroscope coupled with a DS-Ri2 camera. Zerene Stacker (v. 1.04 Build T2020-05-22-1330) software and NIS-Elements-BR (Nikon Imaging Software Basic Research) were used to process and combine multiple photographs. Images were enhanced and arranged in plates in Adobe Photoshop and Illustrator CC 2015.

### Molecular dataset

#### DNA extraction, library preparation and sequencing

Genomic DNA was extracted from an ethanol-preserved female of *H.sicardi* (http://id.luomus.fi/NC.03) following the Qiagen DNeasy Blood & Tissue Kit (QIAGEN). The quality control was performed with a Qubit dsDNA HS (Invitrogen) and Fragment Analyzer (AATI). The generated Nextera Flex library (Illumina) was sequenced using Illumina NextSeq 500 sequencer with the cycles 170-8-8-132 that yielded the lowest coverage genome of *H.sicardi*.

#### Genome assembly and annotation

The read quality was checked with FastQC (Andrews 2010) and adapters were removed using Trimmomatic (Bolger et al. 2014). The trimmed reads were mapped against the reference mitogenome of *H.quadripunctatus* (accession number KU739489) using BWA software and its bwa-mem algorithm (Li and Durbin 2009). This allowed us to assemble ~85% of *H.sicardi* mitogenome (accession number: MW759025) used in the downstream analyses. The assembled mitogenome was annotated in Geneious using the reference mitogenome of *H.quadripunctatus* (Olivier, 1789).

#### Molecular dataset

The ingroup consisted of 44 operational taxonomic units (OTUs) belonging to ~30 species of *Helictopleurus*; two *H.sicardi*OTUs were used, the new one and the one from previous phylogenetic study (Wirta et al., 2008). The outgroup included nine species from various Afrotropical and Oriental lineages of Oniticellini. The dataset comprised 13 protein-coding and two rRNA genes (16S and 12S) from mitogenome, and two nuclear rRNA genes (18S and 28S). Thus, our molecular dataset included novel sequences for *H.sicardi*, as well as GenBank data for *Helictopleurus* and Oniticellini from mitogenomic (Breeschoten et al. 2016) and individual genes (Wirta et al. 2008; Monaghan et al. 2009; see Suppl. material 1) phylogenetic studies. The dataset was compiled using phylotaR (Bennett et al. 2018) and AnnotationBustR (Borstein and O’Meara 2018).

### Phylogenetic analyses

Gene fragments were individually aligned using MAFFT (Katoh et al. 2002) and concatenated into five prior partitions: three codon partitions, mitochondrial rRNA and nuclear rRNA. The best partitioning scheme and substitution model was selected using ModelFinder (Lanfear et al. 2012) implemented in IQ-TREE (Nguyen et al. 2015) under Bayesian Information Criterion; the best-found scheme matched the prior one. The ModelFinder results were used in the subsequent IQ-TREE search to infer the maximum likelihood (ML) tree. The support values (i.e., bootstrap support, BS) were calculated using ultrafast bootstrap approximation (Minh et al. 2013).

## Results and discussion

### Phylogenetic analyses

**Figure 1.**
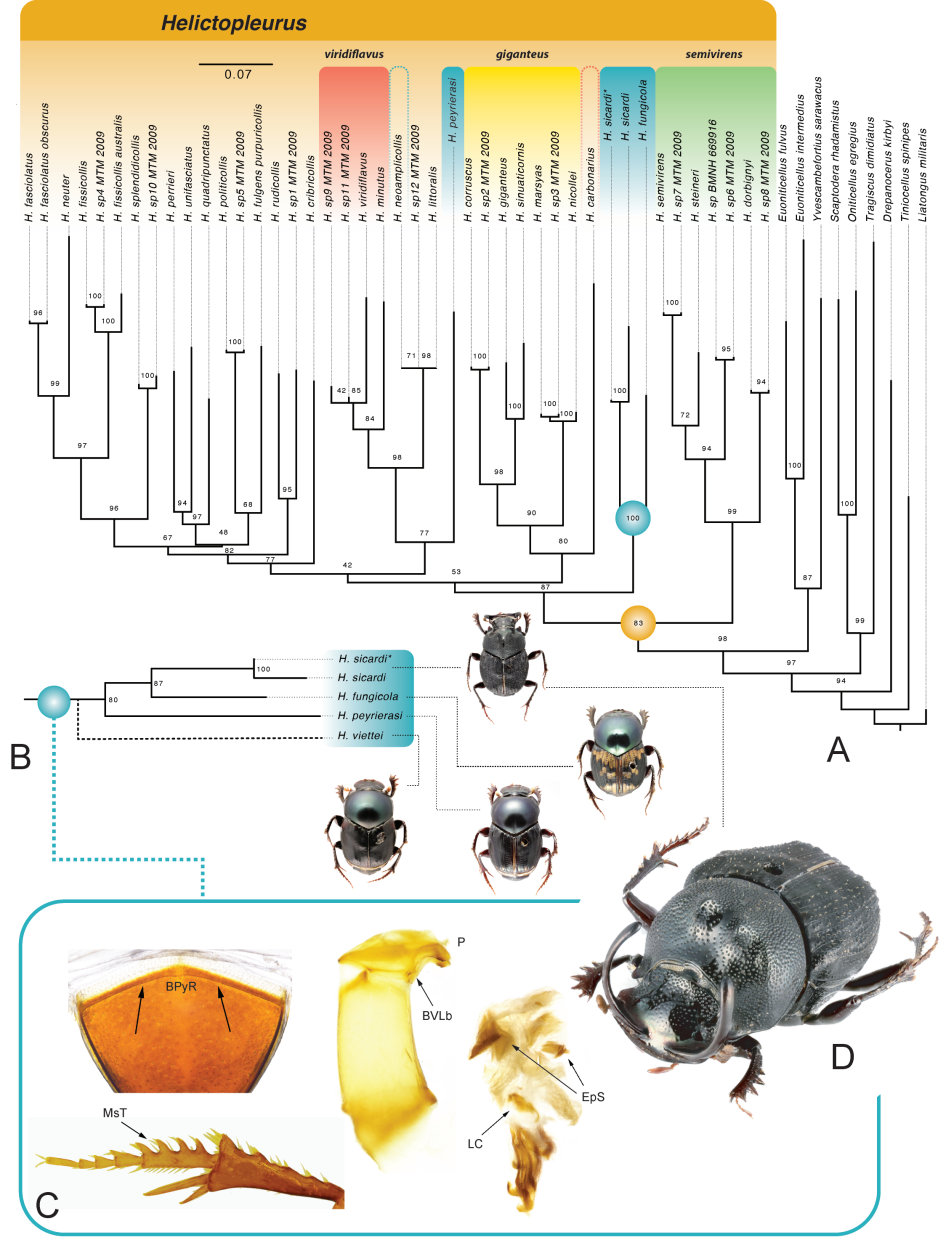
Molecular phylogenies and morphological synapomorphies of the *fungicola* species group **A** phylogenetic position of *H.sicardi* within *Helictopleurus*. *Helictopleurusneoamplicollis* and *H.carbonarius* are highlighted with colored dashed line to indicate their previous placement **B** COI-based phylogeny: magnification of the *fungicola* clade and phylogenetic position of *H.peyrierasi* stat. rest. **C** synapomorphies of the *fungicola* species group: basal pygidial ridge (BPyR); mesotarsal teeth (MsT) on the first tarsomere; parameres (P) elongated and evenly curved downward, basoventral lobes of the parameres bell shaped (BVLb); superior side of the membranous sac of the endophallus with spines (EpS); lamella copulatrix very simple (LC) **D** detail of the clypeal horns of the male of *H.sicardi*.

The combined phylogenetic analyses of the fragments of 19 mitochondrial and nuclear genes support the monophyly of the genus *Helictopleurus* (BS 83) (Fig. 1A). The recovered relationships among Oniticellini genera are consistent with the mitogenomic study of Breeschoten et al. (2016). *Helictopleurussicardi* (both OTUs used) is nested within *Helictopleurus* as the sister species to *H.fungicola* (BS 100). These findings are also supported by the earlier 5-gene phylogenetic analysis of *Helictopleurus* (Wirta et al. 2008). The clade *sicardi+fungicola* and its sister (BS 87) together form sister to the *semivirens* clade and define the first divergence event within the genus.

Interestingly, our combined analysis and that of Wirta et al. (2008) place another member of *fungicola* species group, *H.peyrierasi* stat. rest. (see discussion below), as sister of the *viridiflavus* clade (BS 77, Fig. 1A). However, morphological synapomorphies (see below) and a separate analysis using only COI support the position of *H.peyrierasi* as the sister to *sicardi+fungicola* clade (BS 80) (Fig. 1B). In our and Wirta et al.’s (2008) analyses, only three gene fragments (COI, 28S, 16S) were available for *H.peyrierasi*. We believe that the placement of *H.peyrierasi* as sister of the clade *viridiflavus* is an artefact of the data deficiency. Following the morphological evidence and COI results, we continue to treat *H.peyrierasi* as a member of the *fungicola* group, while *H.neoamplicollis* is excluded from it.

### Systematics and diagnosis of the *fungicola* species group

According to Montreuil (2005a), the *fungicola* group includes the following six species: *H.fungicola*, *H.peyrierasi* stat. rest. (see discussion below), *H.viettei* Paulian & Cambefort, 1984, *H.pluristriatus* d’Orbigny, 1915, *H.neoamplicollis* Krell, 2000, and *H.nigritulus* Lebis, 1960. Here we examined the morphology of 39 *Helictopleurus* species belonging to all the currently known species groups (*sensu*Montreuil 2005a) to elucidate putative synapomorphies and formulate a new definition of the *fungicola* clade recovered in our molecular analyses. The morphological study suggests that the *fungicola* group consists of four species: *H.sicardi*, *H.fungicola*, *H.peyrierasi*, and *H.viettei*. The monophyly of the group is supported by the following putative synapomorphies that can be equally used as diagnostic characters to define the same group (Fig. 1C): abdominal tergites 7^th^ and 8^th^ separated by a thin and distinct ridge; proximal mesotarsomere with spine-like spurs on the lateral edge; parameres elongated and evenly curved downward at the apex; tip of the parameres outwardly oriented; superior region of the membranous endophallus (internal sac) with elongated or scale-like, symmetrically or non-symmetrically distributed spines; and lamella copulatrix simple if compared to the remaining *Helictopleurus* species and composed of one to two close parts connected by a thin and weakly sclerotized region. *Helictopleurusviettei* is the only species of the *fungicola* group not represented in our molecular analyses, but the external and genital morphology of this species suggests its incorporation in the same group (Fig. 1B).

These putative synapomorphies are not found in *H.nigritulus* or in *H.neoamplicollis*, which were formerly assigned to the *fungicola* group. *Helictopleurusnigritulus* exhibits characters that suggest its membership in the *semivirens* group (e.g., pronotum clearly larger than elytral width, surface of the body polished, with very shallow punctures, and head of the female ogive-shaped, with a transverse and straight carina in the frontoclypeal region). The correct taxonomic placement of *H.neoamplicollis* needs further investigation. *Helictopleuruspluristriatus* is here considered to be a new synonym of *H.fungicola* (see below).

Interestingly, *H.villiersi* Paulian & Cambefort, 1984, which was assigned to the *viridiflavus* group (Montreuil 2005a), has the lateral edge of the proximal mesotarsomere serrate as in the species of the *fungicola* group. However, the phylogenetic position of *H.villiersi* remains uncertain.

### Key to the species of the fungicola group

**Table:** 

1	Pronotal punctation strong and coarse; elytral interstriae granulose; major male with long and widely curved clypeal horns (Fig. 4A–C)	***H.sicardi* (Paulian, 1975)**
–	Pronotal punctation very fine; elytral interstriae without granules; male without horns	**2**
2	Clypeal margin of male and female with two acute teeth at middle; male with a small transversal clypeal carina (Fig. 3A, B)	***H.viettei* Paulian & Cambefort, 1984**
–	Clypeal margin of male and female with three blunt to acute teeth at middle; male without clypeal carina	**3**
3	Frontoclypeal region with a distinct hump, postoccipital margin with a pointed tubercle in the middle (Fig. 2P); external tip of the parameres without a small indentation; basoventral lobes of the parameres big and wide (Fig. 2H, I); superior side of the membranous sac of the endophallus with small to medium-sized, scale-like spines (Fig. 2M, N); lamella copulatrix consisting of two leaf-like parts (Fig. 2J)	***H.peyrierasi* Paulian & Cambefort, 1984 stat. rest.**
–	Frontoclypeal region and postoccipital margin simple to slightly swollen (Fig. 2O); external tip of the parameres with a small indentation; basoventral lobes of the parameres small and narrow (Fig. 2C, D); two patches of the superior side of the membranous sac of the endophallus are covered by long, thick and uprightly oriented spines (Fig. 2K, L); lamella copulatrix consisting of a single leaf-like parts (Fig. 2E)	***H.fungicola* (Fairmaire, 1899)**

#### Helictopleurus fungicola

Taxon classificationAnimaliaColeopteraScarabaeidae

(Fairmaire, 1899)

072B2D47-20E2-5854-BF37-FB9E75A9B4EB

**Figure 2.**
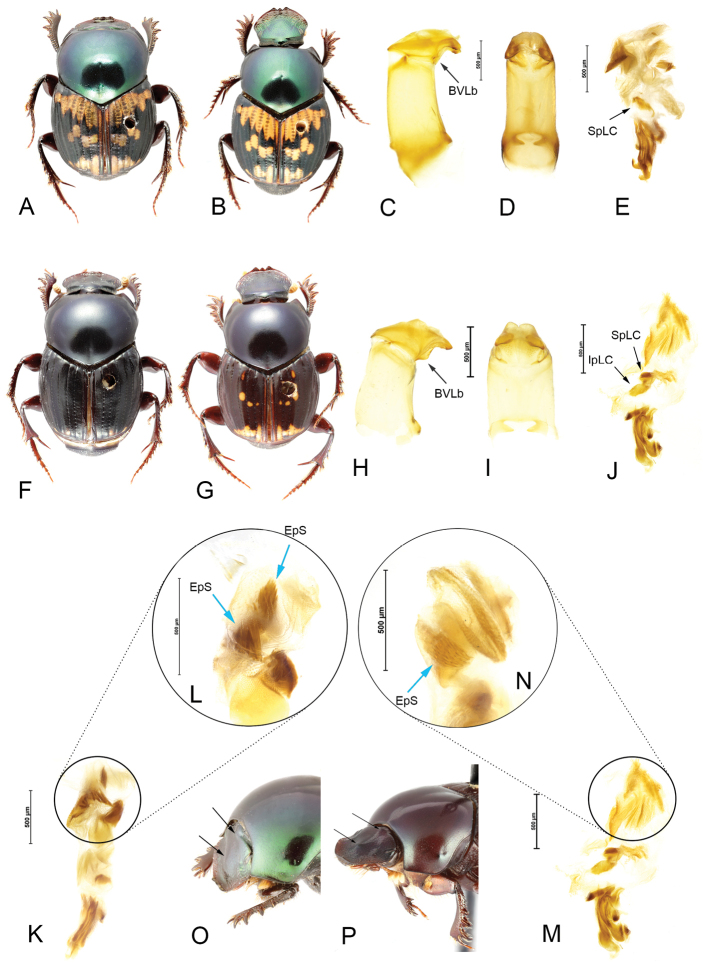
*Helictopleurusfungicola***A, B** habitus of male (**A**) and female (**B**) **C, D** lateral and anterior view of the aedeagus; basoventral lobes (BVLb) **E** endophallus: lamella copulatrix (LC) **K, L** details of the superior side of the membranous sac of the endophallus; Endophallic spines (EpS) **O** lateral view of the head: arrows indicating the absence of humps and tubercles. *Helictopleuruspeyrierasi* stat. rest. **F, G** habitus of male (**F**) and female (**G**) **H, I** lateral and anterior view of the aedeagus **J** endophallus: superior (SpLC) and inferior (IpLC) part of the lamella copulatrix **M, N** details of the superior side of the membranous sac of the endophallus **P** lateral view of the head: arrows indicate the frontoclypeal hump and the postoccipital central tubercle.

Figure 2A–E, K, L, O

Oniticellus fungicola Fairmaire, 1899: 519.
Helictopleurus fungicola : d’Orbigny, 1915: 425; Boucomont and Gillet 1927: 110; Lebis 1960: 97; Paulian 1986: 105; Paulian and Cambefort 1991: 115; Montreuil 2005a: 133; Orsini et al. 2007: 157 (appendix 1); Wirta et al. 2008: 1081 (phylogeny), 1085 (appendix A).
Helictopleurus pluristriatus d’Orbigny, 1915: 426 (syn. nov.); Boucomont and Gillet 1927: 111; Lebis 1960: 102; Paulian 1986: 106; Montreuil 2005a: 133.

##### Type material examined.

Of *H.fungicola*: lectotype, male (here designated): “Madag^r^ Suberb^lle^ H. Perrier / Muséum Paris Madagascar Perrier de la Bathie Coll. L. Fairmaire 1906 / TYPE / Oniticellusfungicola Frm Madag / Oniticellusfungicola Fairmaire, 1899 Rossini et al. des. 2021 / LECTOTYPE / Helictopleurusfungicola (Fairmaire, 1899) Rossini et al. det. 2021” (MNHN).

Of *H.pluristriatus*: holotype, male: “Muséum Paris Madagascar Expéd. La Bonite, Gaudichaud 1837 / pluristriatus n. sp. d’Orb. / HOLOTYPE” (MNHN).

##### Distribution.

This species is distributed from the northernmost region of Madagascar to the central-western coast. It is known from the Diana, Melaky, Boeny, and Menabe regions.

##### Remarks.

**Figure 3.**
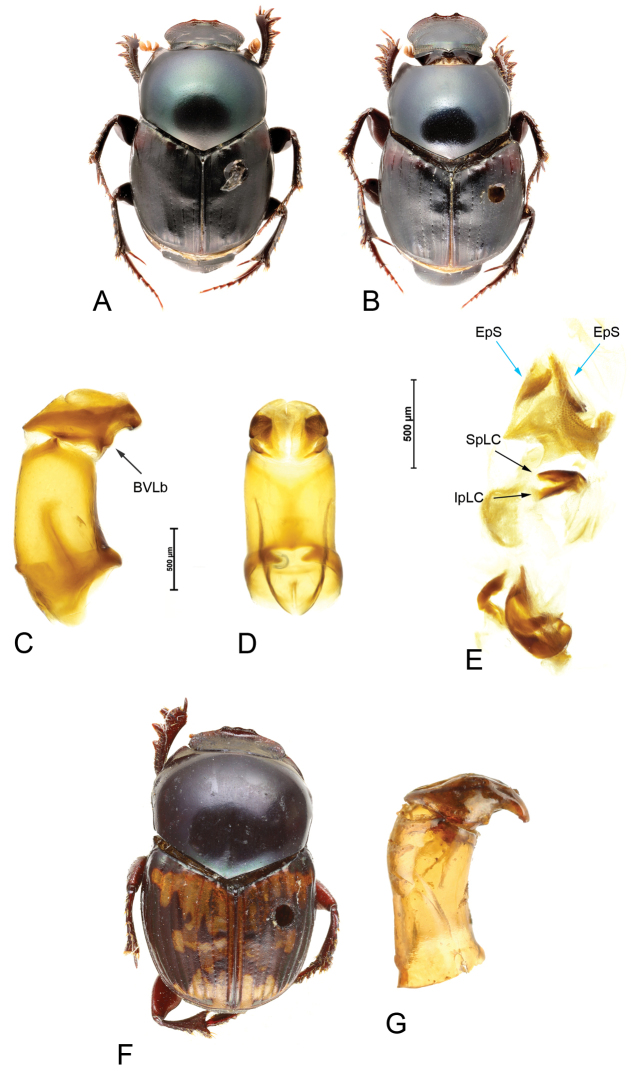
*Helictopleurusviettei***A, B** habitus of male (**A**) and female (**B**) **C, D** lateral and anterior view of the aedeagus; basoventral lobes (BVLb) **E** endophallus: endophallic spines (EpS); superior (SpLC) and inferior (IpLC) part of the lamella copulatrix. *Helictopleuruspluristriatus***F** dorsal habitus of the holotype **G** lateral view of the aedeagus.

The examination of the holotype of *H.pluristriatus* (Fig. 3F, G) revealed that d’Orbigny (1915) described this new *Helictopleurus* using a male of *H.fungicola* with no exact collecting locality. Hence, *H.pluristriatus* syn. nov. is here treated as junior synonym of *H.fungicola* (Fig. 3F, G). *Helictopleuruspluristriatus* was described from a singleton male specimen allegedly collected in Madagascar (locality unknown) by the French botanist C. Gaudichaud-Beaupré during his expedition on board *La Bonite*. All observations, including all the zoological and botanical specimens collected during the expedition were later reported in the *Voyage autour du monde exécuté pendant les années 1836–1837 sur la corvette “La Bonite*”, which was published in 15 volumes (Vaillant 1840–1866; Bousquet 2016). The only stop made by *La Bonite* in the Malagasy region was at Mascarene island, La Réunion. From there the frigate sailed straight toward the Cape of Good Hope (South Africa). Therefore, Gaudichaud could have received the holotype of *H.pluristriatus* from other Madagascar collectors, such as A. Pervillé and J.M.C. Richards with whom he had frequently corresponded and exchanged botanical material. Indeed, nowadays, many of Gaudichaud specimens from Madagascar are thought to have been donated to him by these two French botanists, who were the earliest to have collected natural history specimens in Madagascar. At the moment, we can rule out the possibility that the holotype of *H.pluristriatus* has been collected in La Réunion, as no *Helictopleurus* are today recorded from the island, but just a few introduced *Onthophagus* species (Lacroix and Poussereau 2019). However, its exact collecting locality in Madagascar remains unknown.

#### Helictopleurus peyrierasi

Taxon classificationAnimaliaColeopteraScarabaeidae

Paulian & Cambefort, 1991 stat. rest.

554128C4-B271-515C-8A9F-4A1E42160EF5

Figure 2F–J, M, N, P

Helictopleurus peyrierasi Paulian & Cambefort 1991: 115; Montreuil 2005a: 133.
Helictopleurus fungicola peyrierasi : Montreuil 2005b: 376; Wirta et al. 2008: 1080, 1081 (phylogeny), 1086 (a ppendix A).

##### Type material examined.

***Holotype*,** male: “Madagascar Ouest, réserve spéciale du Zombitsy, Est de Sakaraha, matsabory, 640m, 7-10.II.1974, P. Viette et A. Peyrieras / Holotype Helictopleuruspeyrierasi n. sp. R. Paulian et Y. Cambefort det. 1991 / HOLOTYPE” (MNHN).

***Paratype*,** female: same data as holotype, except the collection date: “13.II.1974” (MNHN).

##### Distribution.

This species is known from the central-western coast of Madagascar (Boeny and Menabe regions).

##### Taxonomic remarks.

Paulian and Cambefort (1991) described *H.peyrierasi* from Zombitsy, south-western Madagascar. Montreuil (2005b), after having examined specimens collected in nearby Kirindy, and having compared them with the type specimens of *Helictopleurusfungicola*, treated this taxon as a subspecies of *H.fungicola.* We compared the type specimens of the nominotypical subspecies with those of *H.fungicolapeyrierasi* and found significant differences, especially in the shape of male genitalia, that support the original treatment of *H.peyrierasi* stat. rest. as a full species within the genus *Helictopleurus*.

#### Helictopleurus viettei

Taxon classificationAnimaliaColeopteraScarabaeidae

Paulian & Cambefort, 1984

71ADC2CF-C311-55DA-8B62-F8D2836E4C72

Figure 3A–E

Helictopleurus viettei Paulian & Cambefort 1984: 113; Montreuil 2005a: 133.

##### Type material examined.

***Holotype*,** female: “Madagascar Ouest, réserve spéciale du Zombitsy, Est de Sakaraha, matsabory, 640m, 13.II.1974, P. viette et A. Peyrieras / Holotype Helictopleurusviettei n. sp. R. Paulian et Y. Cambefort det. 1984 / HOLOTYPE” (MNHN).

***Paratype*,** female: same data as holotype, except the collection date: “7–10.II.1974” (MNHN).

##### Distribution.

Only known from south-western Madagascar (Atsimo-Andrefana region).

#### Helictopleurus sicardi

Taxon classificationAnimaliaColeopteraScarabaeidae

(Paulian, 1975)

35214DD4-6AC9-52B0-A271-E3EF3027DBFD

Figure 4A–I

Heterosyphus sicardi Paulian, 1975: 248; Halffter and Edmonds 1982: 136; Paulian and Cambefort 1984: 50 –51; Paulian 1986: 107; Cambefort 1991: plate 4.6 (unpaginated); Paulian and Cambefort 1991: 113; Davis et al. 2002: 1224; Montreuil 2005a: 134; Wirta et al. 2008: 1080–1081 (caption and phylogenetic tree), 1087 (appendix A); Philips 2011: 27; Sole et al. 2011: 3.
Helictopleurus sicardi : Philips, 2016: 11, 13, 40–41 (synonymy Heterosyphus = Helictopleurus).

##### Type material examined.

***Lectotype*,** female (here designated): “Montagne d’Ambre. I. / Epactoides *nar*? / TYPE / *Heterosyphussicardi* n.g. n.sp. R. Paulian det. /. Heterosyphussicardi Paulian, 1975 des. Rossini et al. 2021 / LECTOTYPE. Helictopleurussicardi (Paulian, 1975) Rossini et al. des. 2021” (MNHN).

***Paralectoype*,** female: “Antsiranana / Madagascar Montagne d’Ambre Muséum Paris Coll. Sicard 1930” (MNHN).

##### Additional material examined.

Madagascar: “Mt. d’Ambre. −12.5281, 49.1709. 1080m. 1.i.2019. sift. MD31. V. Grebennikov, http://id.luomus.fi/NC.01” (1 male MZHF); same data, http://id.luomus.fi/NC.02 (1 female, MZHF; body parts disarticulated); same data, http://id.luomus.fi/NC.03 (1 female MZHF; body parts disarticulated, DNA material); same data (3 males, 7 females, MZHF); “Montagne d´Ambre. Jan 2004. Wet forest. Alt. 1300 m. fish baited trap. Iikka Hanski leg. / http://id.luomus.fi/GZ.19901. I.2004” (1 female, MZHF); same data, “http://id.luomus.fi/GZ.19902. I.2004” (1 male, MZHF).

##### Diagnosis.

Within the endemic Madagascar genus *Helictopleurus*, *H.sicardi* shares a series of morphological characters with the species here assigned to the *fungicola* group. These characters are the posterolateral margin of the pronotum extended in the propleural region with a short ridge beneath the lateral edge of the pronotum; clypeal margin of female with acute teeth at middle (three teeth as in *H.fungicola* and *H.peyrierasi*); parameres elongated; lamella copulatrix very simple and composed by a superior and inferior leaf-like parts; and superior side of the membranous sac of the endophallus with regions covered by scale-like spines. However, *H.sicardi* is easily distinguished from the other members of the *fungicola* group by the large punctation on the pronotum (very fine to absent in *H.fungicola*, *H.peyrierasi*, and *H.viettei*); male with a couple of long and widely curved clypeal horns (head unarmed in the remaining species; with an acute to obtuse post-occipital tubercle in *H.peyrierasi*); body completely brown (pronotum and head dark with blue to emerald green sheen, and elytra bicolored with reddish or yellow spots in the other species of the group); and elytra with rows of bright, elongated granules (granules absent in *H.fungicola*, *H.peyrierasi*, and *H.viettei*).

##### Description of the male.

***Body length and color*.** Body length from clypeal margin to elytral apices 7 mm, dorsal and ventral side of the body brown and bright, dorsal tegument clearly sericeous on the disc of the head, posteromedian region of pronotum and elytral interstriae; mouthparts and antennae light brown, setae light yellow to brownish.

**Figure 4.**
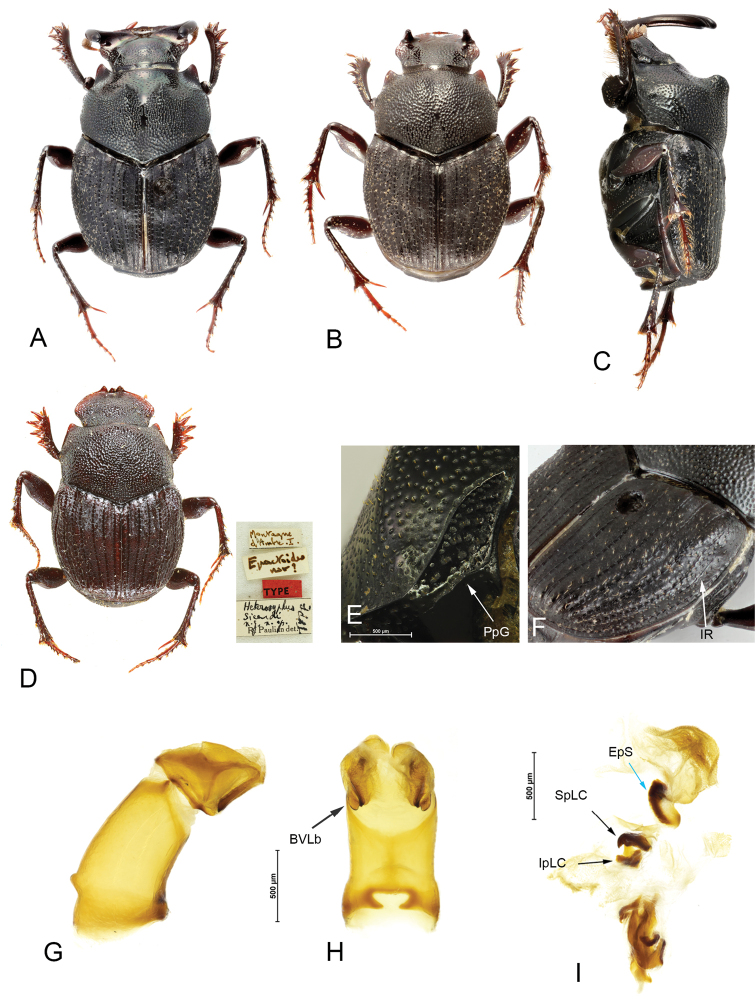
*Helictopleurussicardi***A, B** major (**A**) and minor (**B**) male dorsal habitus **C** major male lateral habitus **D** female dorsal habitus (lectotype) and original labels **E** propleural groove (PpG) **F** VIII interstrial ridge (IR) **G, H** lateral and anterior view of the aedeagus; basoventral lobes (BVLb) **I** endophallus: endophallic spines (EpS); superior (SpLC) and inferior (IpLC) part of the lamella copulatrix.

***Head*.** Clypeus with margin widely and evenly curved, with sides straight and parallel in proximity of the horns, genal margin curved, clypeogenal junction indicated by a short and shallow ridge, and by the base of the clypeal horns. Clypeus with two long and widely curved horns (Fig. 1D, 4A, C), with tips rounded and slightly convergent at middle. Horns laterally flattened and basally strongly widened, bases of the horns occupy most of the lateral region of the clypeus. Clypeus smooth and shiny, clypeal disc with scattered and shallow punctures; frons with punctation coarser and denser. Frons without armature, eye opening very narrow and elongated anteroposteriorly. Antennae with eight articles; antennal club small and rounded.

***Thorax*.** In dorsal view, pronotum narrower with respect to elytra, in lateral view slightly convex. Lateral edges rather straight and weakly divergent from posterior angles to the middle, feebly concave and sinuate from middle to anterior angles. Pronotal anterior angles narrow and obtusely acuminate. Lateral and anterior pronotal edges complete and finely margined, posterior edge with no margin. The pronotal posterior edge is extended in the propleura, beneath the lateral edge of the pronotum, with a shallow groove (Fig. 4E). Anterosuperior region of pronotum with two parallel and high carina, anteriorly oriented and separated by a large depression.

Posteromedial pronotal region with punctation rugulose-lacunose and coarse, central and medial region without punctures, punctation more spaced in the anterior region. Several punctures associated with short and stout setae. Pronotal tegument shiny and smooth on anterior half, posterior half finely microsculptured and especially in the middle.

Propleuron weakly excavated at the bottom of the pronotal anterior angles; propleuron with two carinae, the internal carina thinner and straight, external one stronger and widely sinuate.

Episternum very narrow; mesosternal surface covered by coarse and dense punctures associated with short and stout setae, anterior region of mesosternum with a wide and smooth bead, triangularly pointed backward at middle; metasternum wide and steeply elevated with respect to the mesosternum in its superior region, surface smooth, with fine punctures on the disc and coarse punctures near the mesocoxae.

***Abdomen*.** Elytra with eight glossy striae interrupted by a series of well-spaced and shallow punctures. Interstriae flat, with surface completely microreticulated, interstriae III–VI clearly swollen apically (carinated), interstria VIII with a longitudinal, thin ridge on basal one third (Fig. 4F). All interstriae with one to two rows of bright granules not perfectly aligned and unevenly distributed, each granule bears a short seta bowed backward. Humeral callus well developed, elytral surface with a distinct depression nearby the callus.

Sternites ventrally visible, anterior margin with a double row of coarse and shallow punctures not perfectly aligned, three rows of punctures on the lateral most region of each sternite.

Pygidium flattened, completely margined, pygidial surface finely microreticulated and with scattered, shallow punctures.

***Legs*.** Lateral margin of protibiae with four acute teeth distributed along the anterior half, posterior half serrated, apical and internal margin of protibia with an acute spur directed forward and slightly bent downward; ventral side of protibial with a longitudinal ridge that terminates apically with an acute tooth beneath the superior spur. Meso and metatibiae slender, enlarged apically, and respectively with two and one spiniform spurs at the apex. Profemora elongated, dorsal side smooth, ventral side with coarse and shallow punctures mostly concentrated on posterior half. Mesofemora and metafemora very slender and swollen posteriorly at middle, ventral surface with coarse and shallow punctures on posterior half, fine punctures anteriorly.

First segment of mesotarsi with four spine-like teeth in the external margin, two to three yellow setae inserted between each tooth.

***Morphological variation*.** Minor males either with two small and straight clypeal horns that arise from the sides (Fig. 4B) or without horns, the head is subtrapezoidal, and the anterosuperior pronotal carina are absent; the anterior half of the pronotum is feebly depressed longitudinally at middle.

Females differ from males by the clypeal margin with three teeth distinctly reflexed upward, the lateral teeth obtuse, while the central one more acuminated, posterior margin of the lateral teeth with short setae; head surface covered by coarse and shallow punctures even on the clypeus where the punctation is shallower; pronotum not depressed medially; protibia without internoapical tooth; last abdominal sternite narrower at middle.

***Male genitalia*.** Parameres elongated, ventrally defined by two straight laminas, basoventral side of the parameres with two lateral lobes obtusely squared (BVLb, Fig. 4G, H). Lamella copulatrix simple and consisting of a superior (SpLC) and inferior (IpLC) leaf-like part; margin of the superior part with a sharp hook (Fig. 4I). Superior side of the membranous sac of the endophallus covered by a scale-like spines (EpS, Fig. 4I).

## Supplementary Material

XML Treatment for Helictopleurus fungicola

XML Treatment for Helictopleurus peyrierasi

XML Treatment for Helictopleurus viettei

XML Treatment for Helictopleurus sicardi

## Supplementary materials

Supplementary material 1 DNA sequence alignment of the material examined in our study Authors: Michele Rossini, Olivier Montreuil, Vasily Grebennikov, Sergei Tarasov Data type molecular data Explanation note Copyright notice: This dataset is made available under the Open Database License (http://opendatacommons.org/licenses/odbl/1.0/). The Open Database License (ODbL) is a license agreement intended to allow users to freely share, modify, and use this Dataset while maintaining this same freedom for others, provided that the original source and author(s) are credited. https://binary.pensoft.net/file/534487
